# Fludarabine plus reduced-intensity busulfan versus fludarabine plus myeloablative busulfan in patients with non-Hodgkin lymphoma undergoing allogeneic hematopoietic cell transplantation

**DOI:** 10.1007/s00277-023-05084-x

**Published:** 2023-01-12

**Authors:** Kimimori Kamijo, Yoshimitsu Shimomura, Akihito Shinohara, Shohei Mizuno, Minoru Kanaya, Yoshiaki Usui, Sung-Won Kim, Takahide Ara, Ishikazu Mizuno, Takuro Kuriyama, Hideyuki Nakazawa, Ken-ichi Matsuoka, Shigeru Kusumoto, Nobuo Maseki, Masaki Yamaguchi, Takashi Ashida, Makoto Onizuka, Takahiro Fukuda, Yoshiko Atsuta, Eisei Kondo

**Affiliations:** 1 Department of Hematology, Kobe City Hospital Organization Kobe City Medical Center General Hospital, 2-1-1 Minatojima-Minamimachi, Kobe, Chuo-Ku 650-0047 Japan; 2 Department of Environmental Medicine and Population Science, Graduate School of Medicine, Osaka University, Suita, Japan; 3 Department of Hematology, Tokyo Women’s Medical University, Tokyo, Japan; 4 Division of Hematology, Department of Internal Medicine, Aichi Medical University, Nagakute, Japan; 5 Department of Cancer Immunology, Institute for Cancer Research, Oslo University Hospital, Oslo, Norway; 6 Department of Hematology, Hokkaido University Hospital, Sapporo, Japan; 7 Division of Cancer Information and Control, Department of Preventive Medicine, Aichi Cancer Center, Nagoya, Japan; 8 Department of Hematopoietic Stem Cell Transplantation, National Cancer Center Hospital, Tokyo, Japan; 9 Department of Hematology, Hyogo Cancer Center, Akashi, Japan; 10 Department of Hematology, Hamanomachi Hospital, Fukuoka, Japan; 11 Department of Hematology, Shinshu University School of Medicine, Matsumoto, Japan; 12 Department of Hematology and Oncology, Okayama University Hospital, Okayama, Japan; 13 Department of Hematology and Oncology, Nagoya City University Graduate School of Medical Sciences, Nagoya, Japan; 14 Department of Hematology, Saitama Cancer Center, Saitama, Japan; 15 Department of Hematology, Ishikawa Prefectural Central Hospital, Kanazawa, Japan; 16 Division of Hematology and Rheumatology, Department of Internal Medicine, Kindai University Hospital, Osakasayama, Japan; 17 Department of Hematology and Oncology, Tokai University School of Medicine, Isehara, Japan; 18 Japanese Data Center for Hematopoietic Cell Transplantation, Nagakute, Japan; 19 Department of Registry Science for Transplant and Cellular Therapy, Aichi Medical University School of Medicine, Nagakute, Japan; 20 Department of Hematology, Kawasaki Medical School, Kurashiki, Japan

**Keywords:** Busulfan, Conditioning regimen, Lymphoma, Non-Hodgkin lymphoma, Stem cell transplantation

## Abstract

Allogeneic hematopoietic cell transplantation (HCT) offers a possible cure for patients with relapsed and refractory non-Hodgkin lymphoma (NHL) through potentially beneficial graft versus lymphoma effects. However, allogeneic HCT is associated with high nonrelapse mortality (NRM). Fludarabine with reduced-intensity busulfan (Flu/Bu2) and myeloablative busulfan (Flu/Bu4) are commonly used in conditioning regimens for allogeneic HCT; however, data on their use in patients with NHL is limited. We investigated the effect of busulfan dose on outcomes by comparing Flu/Bu2 and Flu/Bu4 in patients with NHL who underwent allogeneic HCT. Our study included 415 adult patients with NHL who received Flu/Bu2 (315 patients) or Flu/Bu4 (100 patients) between January 2008 and December 2019. All patients were enrolled in the Transplant Registry Unified Management Program 2 of the Japanese Data Center for Hematopoietic Cell Transplantation. The primary endpoint was the 5-year overall survival (OS). To minimize potential confounding factors that may influence outcomes, we performed propensity score matching. The 5-year OS was 50.6% (95% confidence interval (CI), 39.4%–60.8%) and 32.2% (95% CI, 22.4–42.4%) in the Flu/Bu2 and Flu/Bu4 groups, respectively (*p* = 0.006). The hazard ratio comparing the two groups was 2.13 (95% CI, 1.30–3.50; *p* = 0.003). Both groups had a similar 5-year cumulative incidence of relapse (38.2% vs 41.3%; *p* = 0.581), and the Flu/Bu4 group had a higher cumulative incidence of 5-year NRM (15.7% vs 31.9%; *p* = 0.043). In this study, Flu/Bu4 was associated with worse OS compared with Flu/Bu2 because of high NRM in patients with NHL.

**Supplementary information:**

The online version contains supplementary material available at 10.1007/s00277-023-05084-x.

## Introduction

Non-Hodgkin lymphoma (NHL) is a heterogeneous group of hematological malignancies with different clinical and histological characteristics [1], and more than 60% of newly diagnosed NHL patients are 60 years or older [2]. Most patients with NHL respond to initial or salvage therapy with autologous hematopoietic cell transplantation (HCT), although patients with relapsed and refractory NHL have a poor prognosis [3].

Allogeneic HCT offers a possible cure for patients with relapsed and refractory NHL through the potential benefits of graft versus lymphoma effects. However, allogeneic HCT is associated with high nonrelapse mortality (NRM) [4]. Myeloablative conditioning (MAC) has been conventionally used as a conditioning regimen for allogeneic HCT; therefore, the indication for allogeneic HCT is limited to younger patients [5]. With the emergence of reduced-intensity conditioning (RIC), access to allogeneic HCT has expanded, even in older patients [5]. A prospective study comparing RIC and MAC in patients with acute myeloid leukemia showed that MAC has a higher risk of NRM and a lower risk of relapse compared with that of RIC, and overall survival (OS) was higher in MAC [6]. No prospective randomized controlled trials comparing RIC and MAC have been conducted in allogeneic HCT for NHL; therefore, the optimal conditioning regimen continues to be explored [7].

Fludarabine with reduced-intensity busulfan (Flu/Bu2) and fludarabine with myeloablative busulfan (Flu/Bu4) are commonly used in RIC and MAC, respectively [8–11]. Several studies have compared the use of Flu/Bu2 and Flu/Bu4 in the treatment of patients with hematological malignancies, especially acute myeloid leukemia; however, scarce data is available on their use in patients with NHL [8–14].

Therefore, we aimed to investigate the effect of busulfan dose on outcomes by comparing Flu/Bu2 and Flu/Bu4 in patients with NHL who underwent allogeneic HCT using registry data from the Japanese Data Center for Hematopoietic Cell Transplantation (JDCHCT).

## Materials and methods

### Data source and patient selection

**Fig. 1:**
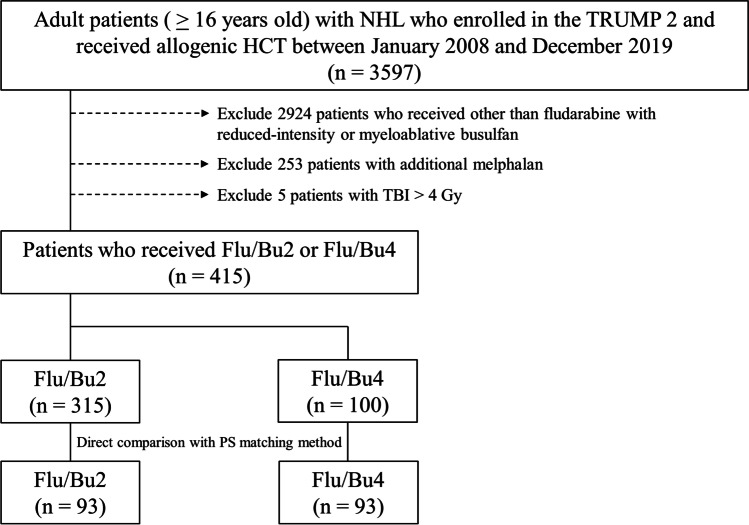
Flow chart of patient selection strategy. Flu/Bu2, fludarabine with reduced-intensity busulfan; Flu/Bu4, fludarabine with myeloablative busulfan; HCT, hematopoietic cell transplantation; NHL, non-Hodgkin lymphoma; PS matching, propensity score matching; TBI, total body irradiation; TRUMP 2, Transplant Registry Unified Management Program 2

Our study included 415 adult patients with NHL who received Flu/Bu2 (315 patients) and Flu/Bu4 (100 patients) (Fig. 1). All patients were enrolled in the Transplant Registry Unified Management Program 2 of the JDCHCT [15, 16]. Flu/Bu2 consisted of intravenous doses of fludarabine and busulfan of 125–180 mg/m^2^ and 6.4 mg/kg, respectively, while Flu/Bu4 consisted of intravenous doses of fludarabine and busulfan of 125–180 mg/m^2^ and 12.8 mg/kg, respectively. Additional total body irradiation (TBI) at a low dose (≤ 4 Gy) was permitted.

Among 3597 patients with NHL aged ≥ 16 years who underwent an initial allogeneic HCT between January 2008 and December 2019, we excluded 2924 patients who received conditioning regimen other than fludarabine with reduced-intensity or myeloablative busulfan, 253 patients who received additional melphalan, and 5 patients who received TBI > 4 Gy (Fig. 1).

Written informed consent was obtained from each patient at each institution before Transplant Registry Unified Management Program 2 registration. This study was approved by the Data Management Committee of the Japan Society for Transplantation and Cellular Therapy and the ethics committee of Kobe City Hospital Organization Kobe City Medical Center General Hospital (approval number: zn220316).

### Propensity score matching

To account for potential confounding factors between treatments that may influence outcomes, we calculated the propensity score (PS) using multivariable logistic regression analysis according to a previously reported standard protocol [17]. We selected the covariates based on the results of previous clinical studies in patients with NHL [14, 18, 19], including age, sex (male vs female), performance status according to the Eastern Cooperative Oncology Group (0–1 vs 2–4), HCT-specific comorbidity index (HCT-CI) (0–2 vs ≥ 3), NHL cell type (B cell vs T/natural killer cell), malignancy grade (indolent vs aggressive), disease status at transplant (complete response (CR) vs partial response (PR) vs no response (NR)), source (related bone marrow (BM) or peripheral blood (PB) vs unrelated BM or PB vs cord blood), donor sex (male vs female), antithymocyte globulin administration (yes vs no), TBI administration (yes vs no), prophylaxis of graft versus host disease (GVHD) (tacrolimus-based vs cyclosporine A-based), prior autologous HCT (yes vs no), > three chemotherapy lines before allogeneic HCT (yes vs no), allogeneic HCT < 24 months after diagnosis (yes vs no), and years of allogeneic HCT (2008–2011 vs 2012–2015 vs 2016–2019). PS matching was applied using the nearest neighbor matching method with calipers of width equal to 0.2. The covariate balances were checked by comparing the standard mean differences between the two groups and were considered to be a negligible imbalance when below 0.25 [20].

### Endpoints and statistical analysis

The primary endpoint of this study was 5-year OS. Secondary endpoints were 5-year progression-free survival (PFS); 5-year cumulative incidence of NRM, relapse, chronic GVHD, and extensive chronic GVHD; 60-day cumulative incidence of neutrophil engraftment; and 1-year cumulative incidence of platelet engraftment, grade II–IV acute GVHD, and grade III–IV acute GVHD.

OS was defined as the time from allogeneic HCT to death from any cause. Relapse was defined as lymphoma recurrence after CR. Patients, who did not achieve CR after allogeneic HCT, were considered relapsed immediately after allogeneic HCT. PFS was defined as the time from allogeneic HCT to relapse or death from any cause. NRM was defined as death from any cause without relapse. Relapse was analyzed considering NRM as a competing risk, and NRM was analyzed considering relapse as a competing risk. Neutrophil and platelet recovery were defined according to the Center for International Blood and Marrow Transplant Research. Neutrophil recovery was defined as the first of three successive days with an absolute neutrophil count of ≥ 500/µL after the posttransplantation nadir. Platelet recovery was considered on the first of three consecutive days when the platelet count was ≥ 20 000/µL in the absence of platelet transfusion for seven consecutive days. Neutrophil and platelet recovery were analyzed considering death as a competing risk. The diagnosis and clinical grading of acute and chronic GVHD were performed according to established clinical criteria [21, 22] and analyzed considering death as a competing risk.

All variables shown in the table and the figures were retrospectively obtained from the JDCHCT registry database. Missing data were imputed by the single imputation method using the R package “missForest.” [23, 24] Continuous variables were expressed as medians and interquartile ranges (quartiles 1–3), and categorical variables were expressed as counts and percentages. For comparisons between the groups, patients and disease characteristics were compared by the Mann–Whitney *U* test for continuous variables and chi-square test for categorical variables. Event rates of 5-year OS and PFS were estimated using the Kaplan–Meier method with a 95% confidence interval (CI). Event relapse rates, NRM, neutrophil recovery, platelet recovery, and acute and chronic GVHD were estimated using Gray’s method with a 95% CI. Univariate and multivariable analyses were performed to estimate the treatment effects of Flu/Bu4 compared with Flu/Bu2 using Cox proportional hazard models for OS and PFS and Fine and Gray methods for the other endpoints. The endpoints are shown in hazard ratios (HRs) and 95% CIs. We selected the adjusted covariates considering the results from previous clinical studies and included the following indices: age, sex, performance status according to the Eastern Cooperative Oncology Group, HCT-CI, NHL cell type, malignancy grade, disease status at transplant, source, donor sex, antithymocyte globulin administration, TBI administration, GVHD prophylaxis, prior autologous HCT, > three chemotherapy lines before allogeneic HCT, allogeneic HCT < 24 months after diagnosis, and years of allogeneic HCT.

Regarding the PS-matched cohort, we estimated OS and PFS using the Kaplan–Meier method and Fine and Gray methods for the other endpoints. The treatment effects of Flu/Bu4 and Flu/Bu2 were compared using Cox proportional hazard models for OS and PFS and Fine and Gray methods for the other endpoints.

In addition, we performed subgroup analysis using the Cox proportional hazard model to examine the treatment effects of Flu/Bu4 compared with those of Flu/Bu2 in each subgroup and the influence of the interactions between conditioning regimens. Statistical significance was set at* p* < 0.05. All statistical analyses were performed using R software (version 4.1.3; R Foundation for Statistical Computing, Vienna, Austria) and EZR (Saitama Medical Center, Jichi Medical University, Saitama, Japan) [25].

## Results

### Baseline characteristics of the entire cohort

The clinical characteristics of the entire patient cohort are shown in Supplementary Table 1 (Online Resource 1). The median age was 56 years (interquartile range: 49–61), and 243 (59%) patients were male. There were 245 (59%) patients with B cell NHL and 170 (41%) patients with natural killer/T cell NHL. The aggressive disease type was found in 315 (76%) patients. Regarding disease status at transplant, 142 (34%), 97 (23%), and 176 (42%) patients had CR, PR, and NR, respectively. The median follow-up was 15.9 months (range: 0.2–151.9 months). Differences were observed in the clinical characteristics between the two groups regarding TBI administration, source, and years of allogeneic HCT. More patients were administered TBI in the Flu/Bu2 than in the Flu/Bu4 group (63% versus 46%; *p* = 0.003). More patients received cord blood transplantation in the Flu/Bu4 than in the Flu/Bu2 group (19% versus 6.7%; *p* = 0.001). Regarding years of allogeneic HCT, more patients in the Flu/Bu2 group underwent allogeneic HCT from 2008 to 2011, whereas more patients in the Flu/Bu4 group underwent allogeneic HCT from 2012 to 2015. After PS matching, 93 patients were included in both groups. The clinical characteristics after PS matching are shown in Table 1. The patient characteristics were well balanced with PS matching, and no significant differences were observed in TBI administration, source, and years of allogeneic HCT between groups.

**Table 1:** Patient characteristics after PS matching

	All (*N* = 186)	Flu/Bu2 (*n* = 93)	Flu/Bu4 (*n* = 93)	*p* value	SMD
Age at transplant	55 (47, 60)	55 (48, 59)	54 (47, 60)	0.794	0.006
Male	110 (59%)	56 (60%)	54 (58%)	0.765	0.065
ECOG PS ≥ 2	24 (13%)	13 (14%)	11 (12%)	0.662	0.064
HCT-CI ≥ 3	28 (15%)	15 (16%)	13 (14%)	0.682	0.060
NHL cell type				0.763	0.044
B cell	114 (61%)	56 (60%)	58 (62%)		
T/NK cell	72 (39%)	37 (40%)	35 (38%)		
Disease type				1.000	< 0.001
Indolent	40 (22%)	20 (22%)	20 (22%)		
Aggressive	146 (78%)	73 (78%)	73 (78%)		
Disease status				0.568	0.156
CR	67 (36%)	36 (39%)	31 (33%)		
PR	46 (25%)	24 (26%)	22 (24%)		
NR	73 (39%)	33 (35%)	40 (43%)		
Donor type				0.873	0.077
Related BM or PB	71 (38%)	37 (40%)	34 (37%)		
Unrelated BM or PB	85 (46%)	42 (45%)	43 (46%)		
CB	30 (16%)	14 (15%)	16 (17%)		
Male donor	121 (65%)	59 (63%)	62 (67%)	0.645	0.068
ATG administration	28 (15%)	13 (14%)	15 (16%)	0.682	0.060
TBI administration	91 (49%)	47 (51%)	44 (47%)	0.660	0.065
Tacrolimus-based GVHD prophylaxis	141 (76%)	71 (76%)	70 (75%)	0.864	0.025
Prior autologous HCT	75 (40%)	38 (41%)	37 (40%)	0.881	0.022
> Three chemotherapy lines before allogeneic HCT	74 (40%)	37 (40%)	37 (40%)	1.000	< 0.001
Allogeneic HCT < 24 months after diagnosis	100 (54%)	50 (54%)	50 (54%)	1.000	< 0.001
Years of allogeneic HCT				0.717	0.120
2008–2011	36 (19%)	16 (17%)	20 (22%)		
2012–2015	72 (39%)	38 (41%)	34 (37%)		
2016–2019	78 (42%)	39 (42%)	39 (42%)		

*ATG* antithymocyte globulin, *BM* bone marrow, *CB* cord blood, *CR* complete response, *ECOG PS* performance status according to the Eastern Cooperative Oncology Group, *FluBu2* fludarabine with reduced-intensity busulfan, *FluBu4* fludarabine with myeloablative busulfan, *GVHD* graft versus host disease, *HCT* hematopoietic cell transplantation, *HCT-CI* hematopoietic cell transplantation-specific comorbidity index, *NHL* non-Hodgkin lymphoma, *NK* natural killer, *NR* no response, *PB* peripheral blood, *PR* partial response, *PS* propensity score, *SMD* standardized mean difference, *TBI* total body irradiation

### Primary endpoint

**Fig. 2:**
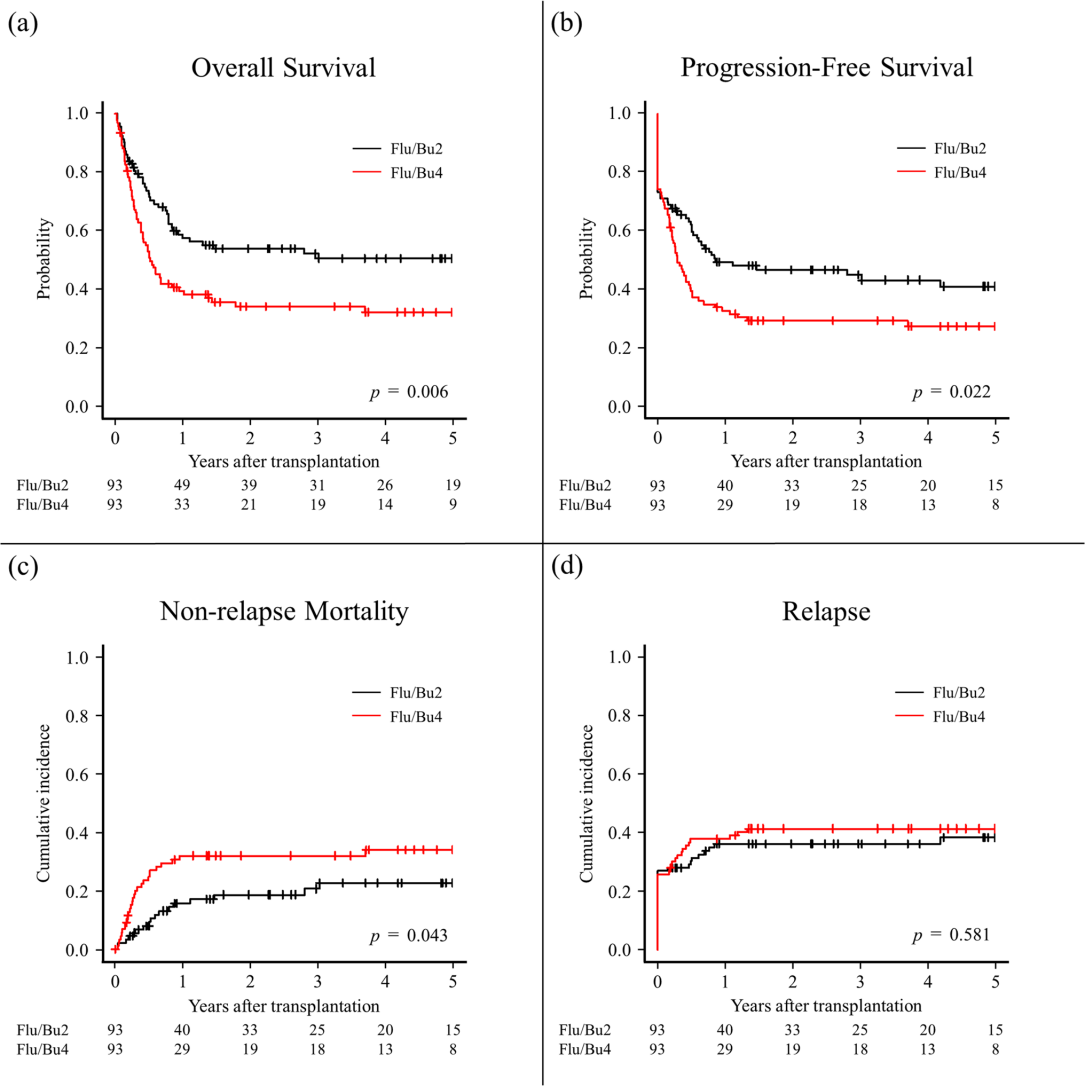
Kaplan–Meier curves of overall survival (**a**), progression-free survival (**b**), cumulative incidence of non-relapse mortality (**c**), and relapse (**d**) after propensity score matching. Flu/Bu2, fludarabine with reduced-intensity busulfan; Flu/Bu4, fludarabine with myeloablative busulfan

Regarding the primary endpoint, the 5-year OS was 55.6% (95% CI, 49.6–61.1) and 31.9% (95% CI, 22.5–41.7) in the Flu/Bu2 and Flu/Bu4 groups, respectively (*p* < 0.001) (Supplementary Fig. 1, Online Resource 1). The HR of OS was 2.10 (95% CI, 1.56–2.84) comparing the Flu/Bu4 and Flu/Bu2 groups (*p* < 0.001). In the multivariable analysis, the adjusted HR was 1.91 (95% CI, 1.36–2.69) comparing the Flu/Bu4 and Flu/Bu2 groups (*p* < 0.001) (Table 2). After PS matching, the 5-year OS was 50.6% (95% CI, 39.4–60.8) and 32.2% (95% CI, 22.4–42.4) in the Flu/Bu2 and Flu/Bu4 groups, respectively (*p* = 0.006) (Fig. 2); the HR was 2.13 (95% CI, 1.30–3.50; *p* = 0.003) (Table 2). The main causes of death were relapse and infection. Thrombotic microangiopathy and veno-occlusive disease were comparable between the Flu/Bu2 and Flu/Bu4 groups (Table 3).

**Table 2:** Hazard ratio of Flu/Bu4 compared with Flu/Bu2

	Univariate analysis			Multivariable analysis			PS matching analysis		
	HR	95% CI	*p* value	HR	95% CI	*p* value	HR	95% CI	*p* value
Overall survival	2.10	1.56–2.84	< 0.001	1.91	1.36–2.69	< 0.001	2.13	1.30–3.50	0.003
Progression-free survival	1.59	1.20–2.10	0.001	1.43	1.05–1.96	0.025	1.40	0.91–2.16	0.128
Nonrelapse mortality	2.02	1.28–3.18	0.002	1.95	1.14–3.34	0.015	1.86	1.03–3.37	0.041
Relapse	1.14	0.83–1.58	0.420	1.14	0.80–1.63	0.460	1.13	0.74–1.71	0.570
Neutrophil engraftment	0.82	0.67–1.01	0.065	0.89	0.69–1.14	0.340	0.92	0.70–1.22	0.550
Platelet engraftment	0.76	0.59–0.97	0.030	0.80	0.58–1.10	0.160	1.01	0.73–1.39	0.960
Grade II–IV acute GVHD	1.22	0.84–1.77	0.290	1.25	0.84–1.88	0.270	1.47	0.92–2.35	0.100
Grade III–IV acute GVHD	1.87	0.95–3.69	0.070	1.67	0.79–3.55	0.180	3.18	1.04–9.75	0.043
Chronic GVHD	0.59	0.37–0.93	0.022	0.62	0.38–1.01	0.050	0.72	0.42–1.25	0.250
Extensive chronic GVHD	0.63	0.36–1.08	0.093	0.71	0.38–1.32	0.280	0.85	0.44–1.65	0.630

*CI* confidence interval, *FluBu2* fludarabine with reduced-intensity busulfan, *FluBu4* fludarabine with myeloablative busulfan, *GVHD* graft versus host disease, *HR* hazard ratio, *PS* propensity score

**Table 3:** Cause of death in propensity score-matched cohort

	Flu/Bu2 (*n* = 93)	Flu/Bu4 (*n* = 93)
Relapse	24	23
Infection	8	15
TMA/VOD	4	4
Acute GVHD	1	5
Chronic GVHD	1	0
Graft failure	0	1
Bleeding	2	1
MOF	1	7
Second cancer	1	0
Others	3	5
Total	45	61

*Flu/Bu2* fludarabine with reduced-intensity busulfan, *Flu/Bu4* fludarabine with myeloablative busulfan, *GVHD* graft versus host disease, *MOF* multiple organ failure, *TMA* thrombotic microangiopathy, *VOD* veno-occlusive disease

### Secondary endpoint

**Fig. 3:**
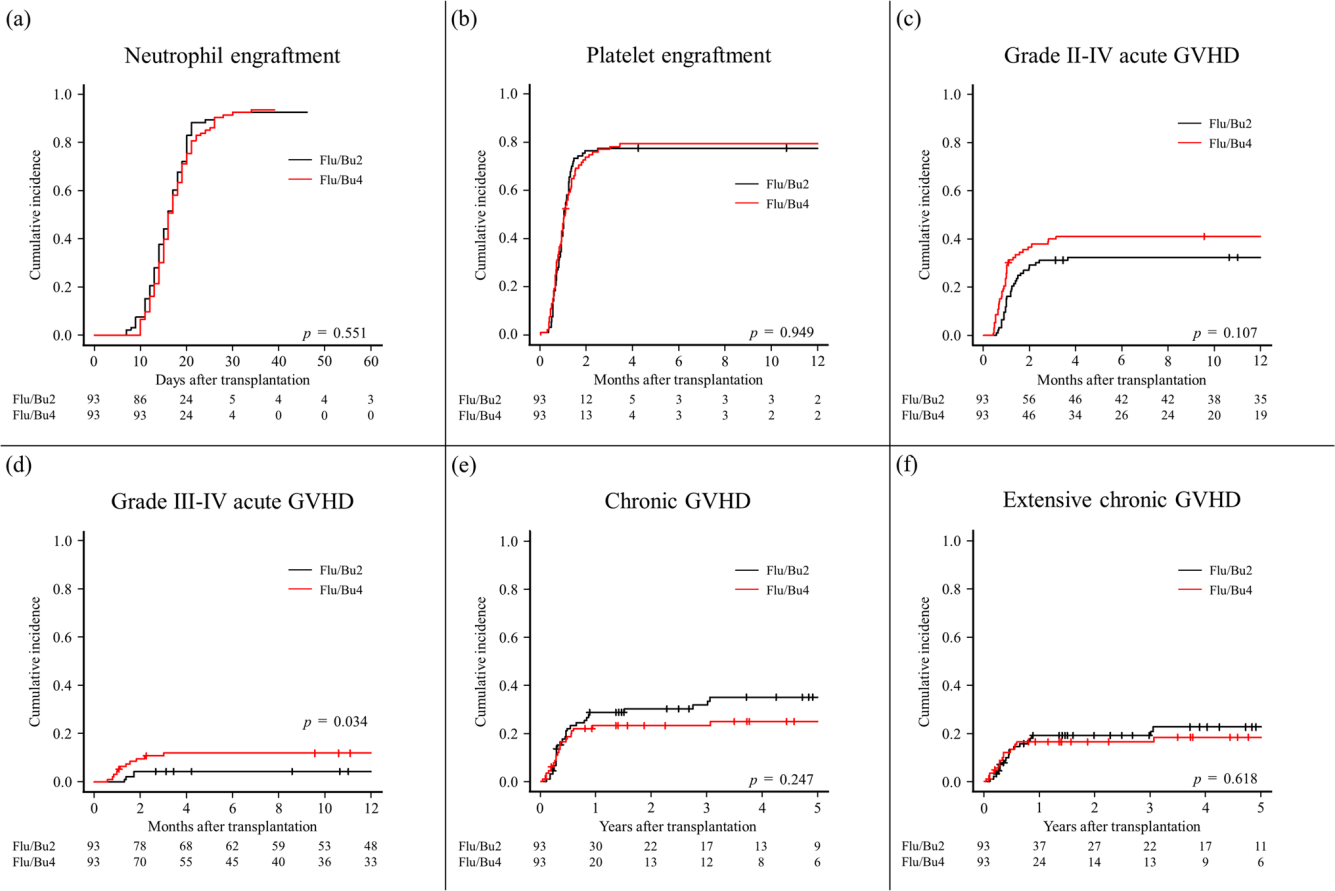
Cumulative incidence of neutrophil engraftment (**a**), platelet engraftment (**b**), grade II-IV acute GVHD (**c**), grade III-IV acute GVHD (**d**), chronic GVHD (**e**), and extensive chronic GVHD (**f**) after propensity score matching. Flu/Bu2, fludarabine with reduced-intensity busulfan; Flu/Bu4, fludarabine with myeloablative busulfan; GVHD, graft-versus-host disease

Regarding the secondary endpoints in the PS-matched cohort, the 5-year PFS was 41.1% (95% CI, 30.0–51.7) and 27.5% (95% CI, 18.5–37.2) in the Flu/Bu2 and Flu/Bu4 groups, respectively (*p* = 0.02). The 5-year cumulative incidence rates of NRM were 22.6% (95% CI, 13.7–33.0) and 33.9% (95% CI, 23.7–44.3) in the Flu/Bu2 and Flu/Bu4 groups, respectively (*p* = 0.043). The 5-year cumulative incidence rates of relapse were 38.2% (95% CI, 27.8–48.5) and 41.3% (95% CI, 31.1–51.2) in the Flu/Bu2 and Flu/Bu4 groups, respectively (*p* = 0.581) (Fig. 2). The 60-day cumulative incidence rates of neutrophil engraftment were 92.5% (95% CI, 84.4–96.4) and 93.5% (95% CI, 86.2–97.0) (*p* = 0.551), and the 1-year cumulative incidence rates of platelet engraftment were 77.4% (95% CI, 67.3–84.7) and 79.2% (95% CI, 69.1–86.3) in the Flu/Bu2 and Flu/Bu4 groups, respectively, (*p* = 0.949). The 1-year cumulative incidence rates of grade II–IV acute GVHD were 32.3% (95% CI, 23.0–41.9) and 41.0% (95% CI, 30.9–50.9) in the Flu/Bu2 and Flu/Bu4 groups, respectively (*p* = 0.107). The 1-year cumulative incidence rates of grade III–IV acute GVHD were 4.3% (95% CI, 1.4–9.9) and 11.9% (95% CI, 6.3–19.5) in the Flu/Bu2 and Flu/Bu4 groups, respectively (*p* = 0.034). The 5-year cumulative incidence rates of chronic GVHD were 34.8% (95% CI, 24.7–45.2) and 24.9% (95% CI, 16.2–34.4) in the Flu/Bu2 and Flu/Bu4 groups, respectively (*p* = 0.247). The 5-year cumulative incidence rates of extensive chronic GVHD were 22.5% (95% CI, 14.1–32.2) and 18.2% (95% CI, 10.8–27.2) in the Flu/Bu2 and Flu/Bu4 groups, respectively (*p* = 0.618) (Fig. 3). The transplant outcomes of the entire cohort are shown in Supplementary Fig. 1 and Fig. 2 (Online Resource 1).

### Subgroup analyses

**Fig. 4:**
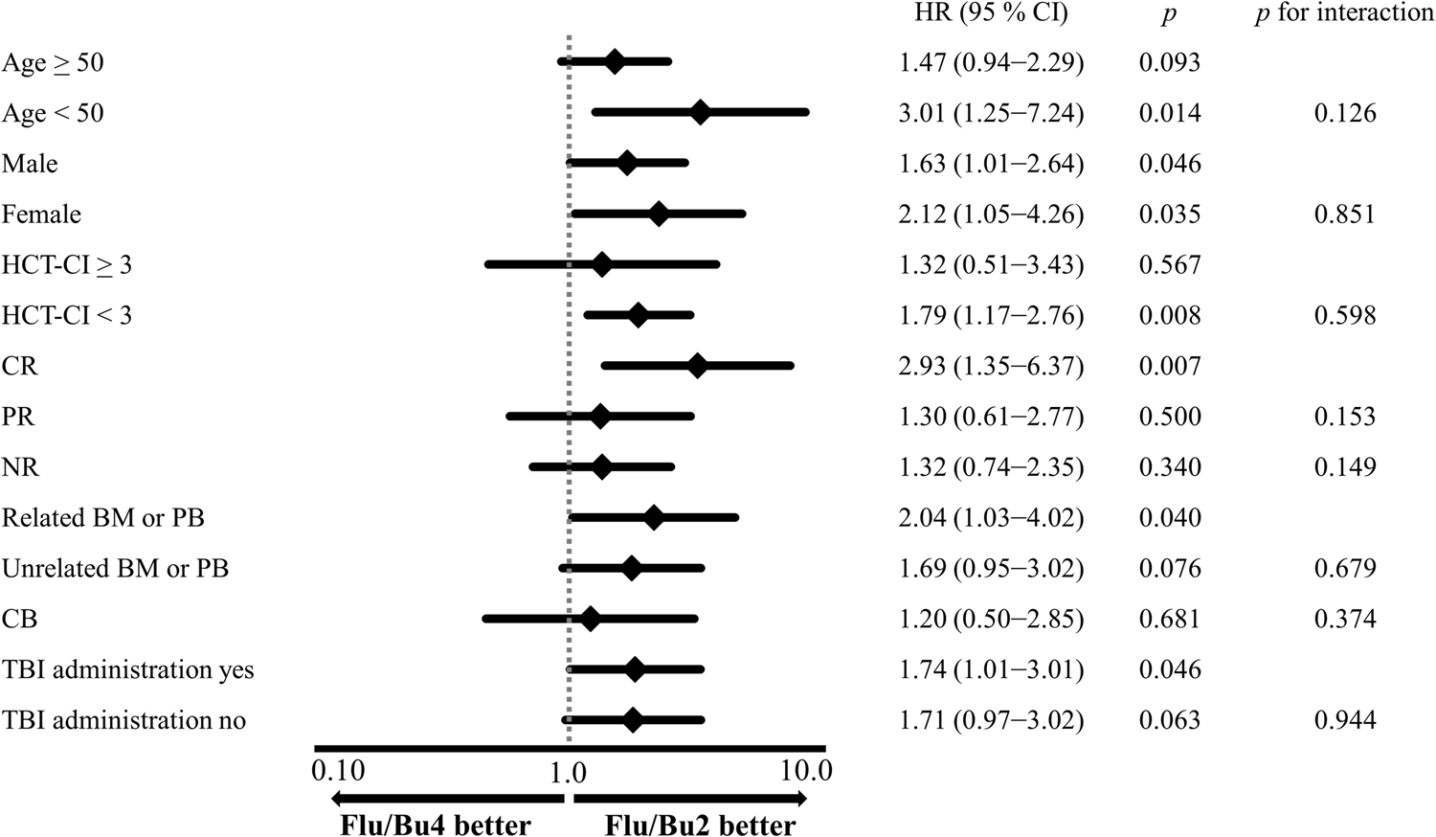
Hazard ratio for Flu/Bu2 compared with Flu/Bu4 for 5-year overall survival in each subgroup. BM, bone marrow; CB, cord blood; CI, confidence interval; CR, complete response; Flu/Bu2, fludarabine with reduced-intensity busulfan; Flu/Bu4, fludarabine with myeloablative busulfan; HCT-CI, hematopoietic cell transplantation-specific comorbidity index; HR, hazard ratio; NR, no response; PB, peripheral blood; PR, partial response; TBI, total body irradiation

To identify patients with more favorable outcomes between the two regimens, we compared outcomes between the two groups after stratification by age, sex, HCT-CI, disease status at allogeneic HCT, source, and TBI. In all subgroups, the OS was favorable with Flu/Bu2 compared with that of Flu/Bu4 (HR: 1.20–3.01). No significant interaction effects were observed between conditioning and age, sex, HCT-CI, PR status, NR status, unrelated BM or PB, CB, and TBI administration (*p* for interaction: 0.126, 0.851, 0.598, 0.153, 0.149, 0.679, 0.374, and 0.944, respectively) (Fig. 4).

## Discussion

This retrospective study population consisted of approximately 40% of NR, which are patients with chemo-refractory disease before allogeneic HCT with a median age of approximately 55 years. Among this population, patients who received Flu/Bu4 had significantly lower OS than patients who received Flu/Bu2 (adjusted HR 1.91, 95% CI, 1.36–2.69) because of similar relapse rates and higher NRM. After PS matching, similar results were confirmed by a HR of 2.13 (95% CI, 1.30–3.50). In the subgroup analysis, the OS was favorable in all Flu/Bu2 subgroups. The present study suggests that Flu/Bu2 is an appropriate regimen compared with Flu/Bu4 and that higher busulfan doses do not contribute to lower relapse rates and may even be harmful in this population.

Generally, patients with hematological malignancies transplanted using RIC have similar survival to those transplanted using MAC because the reduction in NRM rates is offset by the increase in relapse rates [26–30]. However, the effect of RIC compared with MAC may vary among diseases. For example, a randomized controlled trial comparing RIC and MAC in patients with acute myeloid leukemia showed a better OS with MAC than in RIC due to lower relapse rates, indicating antitumor effects and improved survival outcomes from increased conditioning intensity [6]. On the other hand, several retrospective studies of patients with myelodysplastic syndromes reported no statistically significant differences in relapse and OS between RIC and MAC [12, 13]. Regarding patients with NHL, RIC has a similar or higher OS compared with that of MAC [14, 18, 29, 31–34]; however, only a few studies have compared the effects of simple antitumor drug volumes with Flu/Bu2 and Flu/Bu4. The largest retrospective study from the European Society for Blood and Marrow Transplantation compared Flu/Bu2 and fludarabine plus a myeloablative dose of busulfan (Flu/Bu3/4) in patients from a cohort consisting of approximately 90% chemosensitive NHL patients [14]. This study demonstrated a trend for worse OS in patients who received Flu/Bu3/4 than that in patients who received Flu/Bu2 (HR 1.47; 95% CI, 0.96–2.24) [14]. In the study, the Flu/Bu3/4 regimen did not contribute to improved relapse rates compared with Flu/Bu2 [14]. In our study, comprising of approximately 40% chemorefractory NHL patients, relapse rates were comparable between the Flu/Bu2 and Flu/Bu4 groups, and OS was significantly higher in the Flu/Bu2 group. In addition, OS was favorable in the Flu/Bu2 group compared with the Flu/Bu4 group regardless of disease status in the subgroup analysis. These results suggest that increasing the busulfan dose did not contribute to improved OS and decreased relapse rates in our study population.

In contrast, age is an important factor related to NRM. Many studies revealed that older age is associated with higher NRM and poor OS [35, 36], with interactions between NRM and OS and conditioning intensity. In particular, MAC had better outcomes for younger patients and worse outcomes for older patients. Our study revealed that OS was significantly higher in the Flu/Bu2 group compared with the Flu/Bu4 group because of lower NRM in both the total population and subgroups, with no interaction. However, our findings should be interpreted with caution as our study included relatively older patients and many patients with comorbidities because of the nature of NHL. Considering that MAC is reportedly associated with higher OS in young patients with Hodgkin lymphoma [37], future studies are required to clarify pretreatment in younger patient groups.

Developing chimeric antigen receptor T cell therapy has remarkably changed the landscape of relapsed and refractory lymphoma, especially diffuse large B cell lymphoma; however, approximately 60% of patients ultimately experience relapse, and the median PFS was only 5.9 months in the ZUMA-1 trial, which confirmed the efficacy of axicabtagene ciloleucel in patients with relapsed and refractory large B cell lymphoma [38–40]. Allogeneic HCT continues to play an important role in patient management; therefore, further developments are needed to improve outcomes, and the results of allogeneic HCT in patients with NHL in this study and previous studies are inadequate [14, 18, 29, 31–34]. As suggested in our study, it may be useless to rely on simply increasing conditioning of patients with NHL. Therefore, developing new treatments is required. Antibody–drug conjugates such as polatuzumab vedotin, bispecific antibodies, and Burton’s tyrosine kinase inhibitors are emerging for relapsed/refractory NHL [41]. Adding these drugs to the conditioning regimen and posttransplant maintenance treatment is an attractive treatment strategy [42].

## Limitations

Our study had several limitations. First, data on chemotherapy regimens administered before allogeneic HCT were not available for all patients. Second, the indications for the Flu/Bu2 and Flu/Bu4 regimens at each center are unclear, and there was likely selection bias in the choice of conditioning which might have influenced the results. Third, pharmacological data for busulfan was not available in this registry-based study. Busulfan is metabolized variably due to individual differences, with pharmacokinetics varying from 7.7% to 38.7% [43]. Several studies have reported that pharmacokinetically guided busulfan dosing is associated with transplant outcomes [43, 44]. Therefore, caution is warranted when interpreting these results. Fourth, although we used multivariable Cox and PS matching analyses, unmeasured confounding factors may have influenced the selection of the conditioning regimen. Nonetheless, we believe that our findings provide valuable information regarding clinical decisions on NHL conditioning regimens.

## Conclusion

In conclusion, our results showed that Flu/Bu4 was associated with worse OS because of significantly higher NRM and had similar relapse rates compared with those of Flu/Bu2 in patients with NHL.

## Supplementary information

Below is the link to the electronic supplementary material.

Supplementary file1 (DOC 586 KB)

## Data Availability

The data that support the findings of this study are available on request from the corresponding author. The data are not publicly available due to privacy or ethical restrictions.
