# Supplementary material for: Fludarabine plus reduced-intensity busulfan versus fludarabine plus myeloablative busulfan in patients with non-Hodgkin lymphoma undergoing allogeneic hematopoietic cell transplantation
Source: Ann Hematol. 2023 Jan 12;102(3):651–61. doi: 10.1007/s00277-023-05084-x (PMC9977852; doi:10.1007/s00277-023-05084-x)
Supplement: Supplementary file 1 — Supplementary file1 (DOC 586 KB) [file 277_2023_5084_MOESM1_ESM.doc]

**Supplementary Information**

Supplementary Table 1. Patient characteristics of the entire cohort.

|  | All  (N = 415) | Flu/Bu2  (n = 315) | Flu/Bu4  (n = 100) | *p*-value | SMD |
| --- | --- | --- | --- | --- | --- |
| Age at transplant | 56 (49, 61) | 56 (50, 61) | 54 (46, 60) | 0.072 | 0.169 |
| Male | 243 (59%) | 186 (59%) | 57 (57%) | 0.717 | 0.088 |
| ECOG PS > 2 | 34 (8.2%) | 23 (7.3%) | 11 (11%) | 0.240 | 0.129 |
| HCT-CI > 3 | 47 (11%) | 31 (9.8%) | 16 (16%) | 0.090 | 0.184 |
| NHL cell type |  |  |  | 0.247 | 0.134 |
| B cell | 245 (59%) | 181 (57%) | 64 (64%) |  |  |
| T/NK cell | 170 (41%) | 134 (43%) | 36 (36%) |  |  |
| Disease type |  |  |  | 0.769 | 0.034 |
| Indolent | 100 (24%) | 77 (24%) | 23 (23%) |  |  |
| Aggressive | 315 (76%) | 238 (76%) | 77 (77%) |  |  |
| Disease Status |  |  |  | 0.840 | 0.068 |
| CR | 142 (34%) | 110 (35%) | 32 (32%) |  |  |
| PR | 97 (23%) | 72 (23%) | 25 (25%) |  |  |
| NR | 176 (42%) | 133 (42%) | 43 (43%) |  |  |
| Donor type |  |  |  | 0.001 | 0.376 |
| Related BM or PB | 165 (40%) | 130 (41%) | 35 (35%) |  |  |
| Unrelated BM or PB | 210 (51%) | 164 (52%) | 46 (46%) |  |  |
| CB | 40 (9.6%) | 21 (6.7%) | 19 (19%) |  |  |
| Male donor | 269 (65%) | 201 (64%) | 68 (68%) | 0.445 | 0.041 |
| ATG administration | 83 (20%) | 68 (22%) | 15 (15%) | 0.151 | 0.171 |
| TBI administration | 244 (59%) | 198 (63%) | 46 (46%) | 0.003 | 0.343 |
| Tacrolimus-based GVHD prophylaxis | 326 (79%) | 251 (80%) | 75 (75%) | 0.320 | 0.112 |
| Prior autologous HCT | 150 (36%) | 108 (34%) | 42 (42%) | 0.162 | 0.159 |
| > Three chemotherapy lines before allogeneic HCT | 165 (40%) | 126 (40%) | 39 (39%) | 0.859 | 0.020 |
| Allogeneic HCT < 24 months after diagnosis | 213 (51%) | 159 (50%) | 54 (54%) | 0.539 | 0.071 |
| Years of allogeneic HCT |  |  |  | < 0.001 | 0.469 |
| 2008-2011 | 149 (36%) | 129 (41%) | 20 (20%) |  |  |
| 2012-2015 | 132 (32%) | 91 (29%) | 41 (41%) |  |  |
| 2016-2019 | 134 (32%) | 95 (30%) | 39 (39%) |  |  |

ATG, antithymocyte globulin administration; BM, bone marrow; CB, cord blood; CR, complete response; ECOG PS, performance status according to the Eastern Cooperative Oncology Group; FluBu2, fludarabine with reduced-intensity busulfan; FluBu4, fludarabine with myeloablative busulfan; GVHD, graft versus host disease; HCT, hematopoietic cell transplantation; HCT-CI, hematopoietic cell transplantation-specific comorbidity index; NHL, non-Hodgkin lymphoma; NK, natural killer; NR, no response; PB, peripheral blood; PR, partial response; Rel, related donor; SMD, standardized mean difference; TBI, total body irradiation; UR, unrelated donor

Supplementary Figure 1. Kaplan-Meier curves of overall survival (a), progression-free survival (b), cumulative incidence of non-relapse mortality (c), and relapse (d) of the entire cohort.


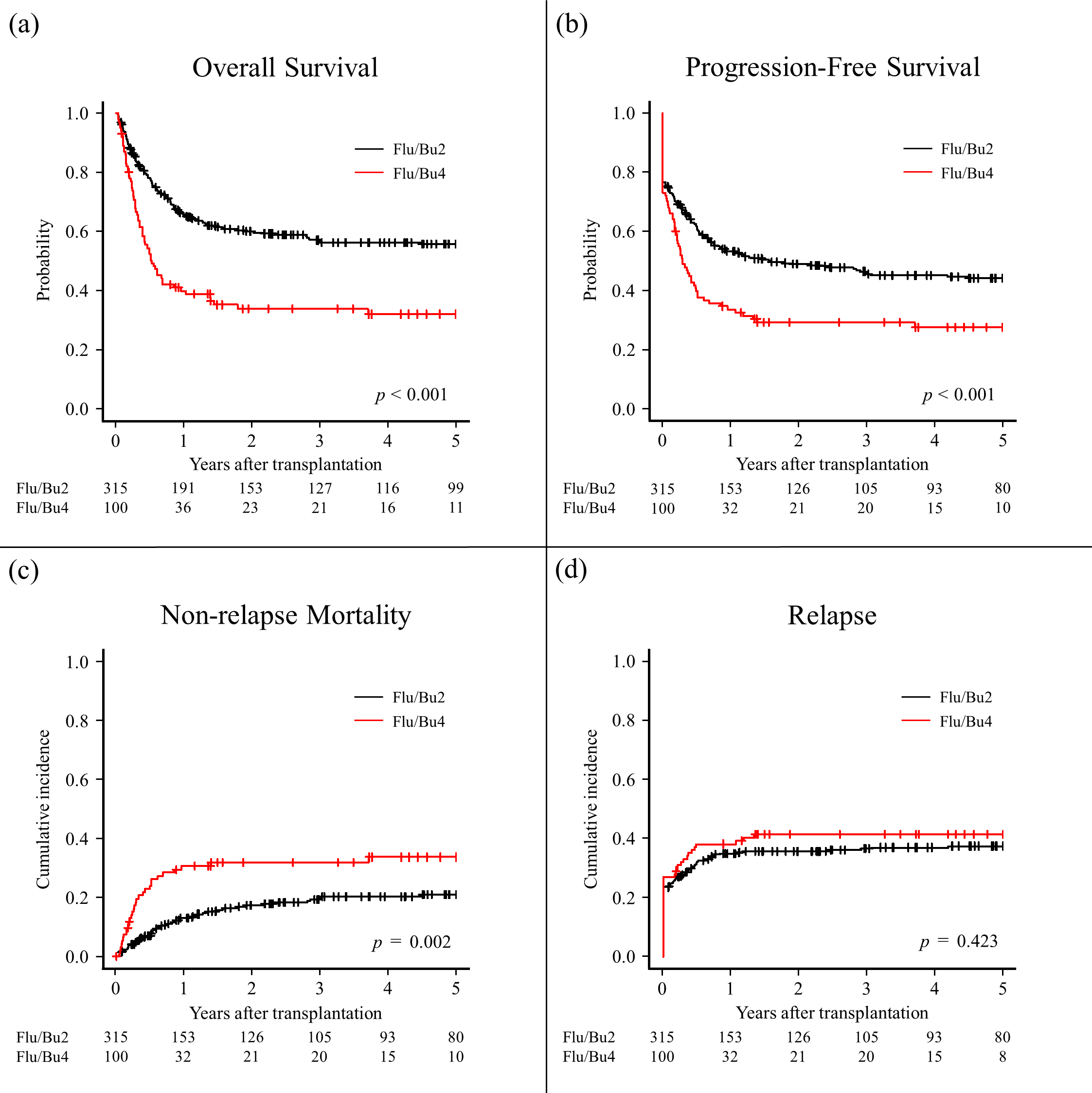


Flu/Bu2, fludarabine with reduced-intensity busulfan; Flu/Bu4, fludarabine with　myeloablative busulfan

Supplementary Figure 2. Cumulative incidence of neutrophil engraftment (a), platelet engraftment (b), grade II-IV acute GVHD (c), grade Ⅲ-IV acute GVHD (d), chronic GVHD (e), and extensive chronic GVHD (f) of the entire cohort.


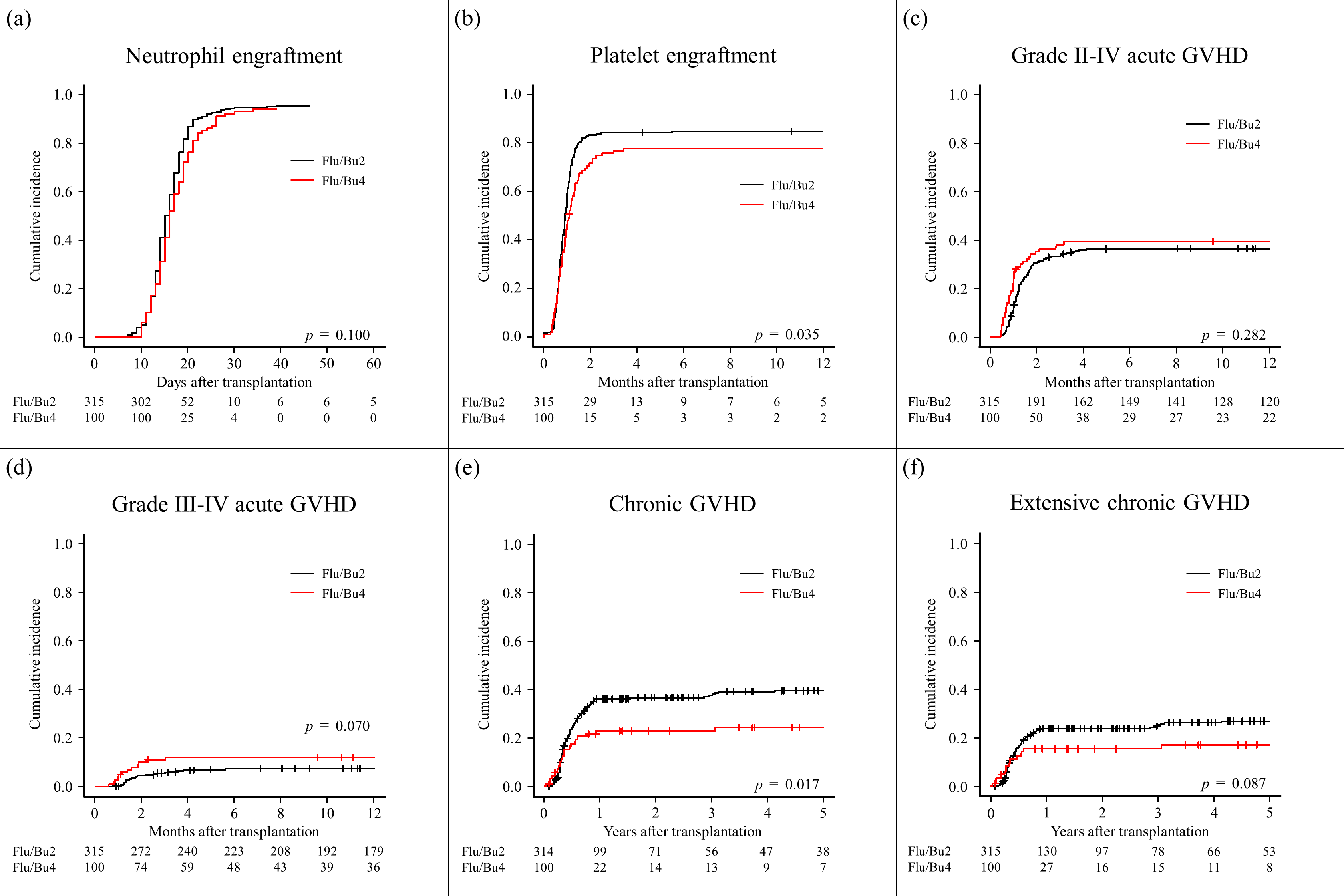


Flu/Bu2, fludarabine with reduced-intensity busulfan; Flu/Bu4, fludarabine with myeloablative busulfan; GVHD, graft-versus-host disease
